# The response of nutrient cycle, microbial community abundance and metabolic function to nitrogen fertilizer in rhizosphere soil of *Phellodendron chinense* Schneid seedlings

**DOI:** 10.3389/fmicb.2023.1302775

**Published:** 2023-12-15

**Authors:** Yuanzheng Gu, Xianglin Chen, Yan Shen, Xiaoyong Chen, Gongxiu He, Xinxing He, Guangjun Wang, Hanjie He, Zhencheng Lv

**Affiliations:** ^1^National Engineering Laboratory for Applied Technology of Forestry & Ecology in South China, Central South University of Forestry and Technology, Changsha, Hunan, China; ^2^Hunan Provincial Key Laboratory of Forestry Biotechnology, Central South University of Forestry and Technology, Changsha, Hunan, China; ^3^International Cooperation Base of Science and Technology Innovation on Forest Resource Biotechnology, Central South University of Forestry and Technology, Changsha, Hunan, China; ^4^College of Arts and Sciences, Governors State University, University Park, IL, United States; ^5^School of Life Sciences, Huizhou University, Huizhou, Guangdong, China

**Keywords:** *Phellodendron chinense* Schneid, nitrogen, soil microorganism, soil metabolism, gene expression

## Abstract

Nitrogen (N) as an essential macronutrient affects the soil nutrient cycle, microbial community abundance, and metabolic function. However, the specific responses of microorganisms and metabolic functions in rhizosphere soil of *Phellodendron chinense* Schneid seedlings to N addition remain unclear. In this study, four treatments (CK, N5, N10 and N15) were conducted, and the soil physicochemical properties, enzyme activities, microbial community abundances and diversities, metabolism, and gene expressions were investigated in rhizosphere soil of *P. chinense* Schneid. The results showed that N addition significantly decreased rhizosphere soil pH, among which the effect of N10 treatment was better. N10 treatment significantly increased the contents of available phosphorus (AP), available potassium (AK), ammonium nitrogen (NH_4_^+^-N), nitrate nitrogen (NO_3_^−^-N) and sucrase (SU) activity, as well as fungal diversity and the relative expression abundances of *amoA* and *phoD* genes in rhizosphere soil, but observably decreased the total phosphorus (TP) content, urease (UR) activity and bacterial diversity, among which the pH, soil organic matter (SOM), AP, NH_4_^+^-N and NO_3_^−^-N were the main environmental factors for affecting rhizosphere soil microbial community structure based on RDA and correlation analyses. Meanwhile, N10 treatment notably enhanced the absolute abundances of the uracil, guanine, indole, prostaglandin F2α and *γ*-glutamylalanine, while reduced the contents of D-phenylalanine and phenylacetylglycine in rhizosphere soil of *P. chinense* Schneid seedlings. Furthermore, the soil available nutrients represented a significant correlation with soil metabolites and dominant microorganisms, suggesting that N10 addition effectively regulated microbial community abundance and metabolic functions by enhancing nutrient cycle in the rhizosphere soil of *P. chinense* Schneid seedlings.

## 1 Introduction

The continuous increase in nitrogen (N) deposition resulting from various human activities, such as industry, agriculture, and livestock farming, has emerged as a significant driver of global environmental change, impacting both forest and soil ecosystems (Peng et al., 2017; He et al., 2023; Huang et al., 2023). Within the soil ecosystem, N input affects the soil physicochemical property, enzyme activity and the relative abundance of gene expression (Cardinale et al., 2020; Chen et al., 2021; Liu et al., 2022; Zhang X. et al., 2022). Previous studies have demonstrated that N addition significantly raised total nitrogen (TN) and available nitrogen (AN) levels, while reducing pH due to increase hydrogen proton concentration, resulting in soil acidification and promoting soil organic carbon (SOC) degradation in the rhizosphere soil of wheat fields and *Cunninghamia lanceolate* forests (Liu et al., 2022; Xu et al., 2022). Interestingly, N application shows no evident impact on the TN and total organic carbon (TOC) contents, whereas enhances the activities of urease and acid phosphatase in soil of bamboo and secondary evergreen broad-leaves forests (Tu et al., 2014; Peng et al., 2017). In addition, N addition significantly increases the abundances of soil *nirK*, *nirS, narG* and *ppx* genes, while distinctly decreases the soil *nifH* abundance in *Metasequoia glyptostroboides* plantations (Wang et al., 2022). However, N addition does not significantly affect the abundances of soil N and P cycling genes in the Alpine Meadow (Xiao et al., 2022). Therefore, N input plays a crucial role in regulating soil physicochemical properties, enzyme activities and gene expressions, with outcomes depending on factors such as plant types, N concentration and agrotype (Tu et al., 2014; Peng et al., 2017; Xu et al., 2022).

N input not only regulates soil physicochemical property and enzyme activity, but also exerts a profound influence on the proliferation, diversity, community composition, relative abundance and metabolic function of soil microorganisms (Ma et al., 2021; Si et al., 2022; Zhang X. et al., 2022). N addition has been reported to reduce the soil bacterial diversity, with significant correlation with physicochemical properties, and it also leads to increase relative abundances of *Proteobacteria* and *Actinobacteria* (Dai et al., 2018; Zhang X. et al., 2022). In addition, N application significantly enhances the fungal diversity and relative abundances of certain fungi, showing significant correlations with soil available nutrients, but it does not appear to impact fungal community composition in tropical/subtropical forests, suggesting that N input primarily influences microbial diversity and relative abundance by modulating soil physicochemical properties (He et al., 2021, 2022; Zhang X. et al., 2022). At the same time, N input influences the contents of proteins, amino acids, nucleic acids, and hormones containing N elements, all of which play pivotal roles in various metabolic processes in soil. These findings underscore the capacity of N addition to affect the metabolic functions of microorganisms in the rhizosphere soil of plants (Cheng et al., 2022).

The *P. chinense* Schneid is an important traditional medicinal plant which mainly distributed in the elevation range of 800–2000 meters in subtropical evergreen broad-leaved forests of China (He et al., 2020; Zhang et al., 2023). The stem and root barks of this plant are rich in various bioactive compounds such as berberine and phellodendrine, which widely uses for the clinic therapies of cancer, hepatitis, pneumonia, diabete and arthritis (Sun et al., 2019; Rauf et al., 2021; Nematollahi et al., 2022). We have demonstrated that N addition increased the chlorophyll content, photosynthetic efficiency, N metabolic capacity, berberine content and relative expression level of *TETRAHYDROPROTOBERBERINE OXIDASE* gene, implying that N addition promoted the growth and berberine synthesis of *P. chinense* Schneid by improving the N metabolic capacity. However, the effects of N addition on physicochemical property, enzyme activity, gene expression, microbial community composition, microbial relative abundance and metabolic function in rhizosphere soil of *P. chinense* Schneid seedlings are still unclear. Therefore, this study aimed to investigate the response mechanism of microorganisms and metabolic functions to N addition in rhizosphere soil of *P. chinense* Schneid seedlings.

## 2 Materials and methods

### 2.1 Study site

The experiment was conducted at the campus practical field of the Central South University of Forestry and Technology, Changsha City, Hunan Province, China (112.9902°E, 28.1319°N). It is a subtropical monsoon humid climate area with the following average annual values: temperature is 17.2°C, precipitation is 1361.6 mm, frost-free period is 275 days, and sunshine is 1529.3 h. We collected the 10 cm deep soil of experimental field using a completely randomized method before the experiment. The soil samples were sieved through a mesh (2 mm) and used to determine the basic physicochemical properties after drying at room temperature. The experimental soil is laterite which basic physicochemical properties are listed as follows: the pH value is 6.35, the SOM content is 24.28 g kg^−1^, the TN content is 1.58 g kg^−1^, and the TP content is 0.68 g kg^−1^ in the plough layer (0–20 cm).

### 2.2 Experimental design

The seeds of *P. chinense* Schneid were collected from a forest farm in Xiangxi, Hunan province, China. The experiment was performed in April 2020. After germination and cultivation, healthy and vital *P. chinense* Schneid seedlings (plant height ≥ 20 cm) were transplanted to the experimental field, with planting row spacing of 1.0 m × 1.0 m. The seedlings were continuously cultivated in the field and managed by manual weeding and watering. After two-months cultivation, the N (urea, Sinopharm, Shanghai, CHN) addition treatment was performed when the seedlings reached about 40 cm height.

In this study, four N concentration treatments were selected for *P. chinense* Schneid seedlings which included: 0 g m^−2^ (CK), 5 g m^−2^ (N5), 10 g m^−2^ (N10) and 15 g m^−2^ (N15). Each treatment contained six seedlings and the experiment were repeated triplicates. N fertilizers were applied around the seedlings in a circular and uniform manner. The experimental fields were subjected to regular management practices, including manual weeding and watering. After the N application treatment 60 days, we performed the destructive sampling of each group. We took out the roots of plant after loosening the soil, shook off the soil tightly combined with the root and brushed them from the root as rhizosphere soil samples. The soil samples were collected from the rhizosphere soils corresponding to each N application treatment and were sieved through a mesh (2 mm) before testing. The treated soil samples were divided into three portions: the first part was used to determine the soil physicochemical properties after drying at room temperature, the second portion was used for analyzing enzyme activities and the third part was saved at −80°C for high throughput sequencing of microorganisms and ultra-high performance liquid chromatography-tandem mass-spectrometry analysis of metabolism.

### 2.3 Soil physicochemical property measurement

The determination of soil pH was using a pH meter at soil-to-water ratio of 1:2.5 (Zhao et al., 2022). The SOM content was determined by potassium dichromate (Sun et al., 2020). The determination of TN was using semi-micro Kjeldahl method (Ma et al., 2021). The determinations of ammonium N (NH_4_^+^-N) and nitrate N (NO_3_^−^-N) contents were using the continuous flow analyzer (SAN++, Skalar Analytical B.V, Breda, NED) (Wang X. et al., 2021). The determinations of total phosphorus and available phosphorus were using the molybdenum antimony colorimetric method (Ma et al., 2021). The determination of available potassium (AK) was using an inductively coupled plasma spectrometer (ICP-AES, Thermo Fisher, CA, USA) (Zhao et al., 2022).

### 2.4 Rhizosphere soil enzyme activity measurement

The determination of soil urease (UR) was using sodium hypochlorite colorimetric method (Sun et al., 2020). The catalase (CAT) activity was determined by potassium dichromate (Li et al., 2022). The determination of sucrase (SU) was using 3, 5-dinitrosalicylic acid (DNSA) method with sucrose used as a substrate and acid phosphatase (ACP) activity was assayed by the p-nitrophenyl phosphate disodium method (PNPP) at pH 6.5 (Sun et al., 2020).

### 2.5 Microbial community composition and diversity analysis

The DNA from 0.50 g soil samples was extracted using PowerSoil® DNA Isolation Kit (12888-50, MOBio, CA, USA) and stored at −80°C for sequencing analysis. For microbial community and diversity analysis, the V4 + V5 region of bacterial 16S rDNA gene was amplified with primers 515F (5′-GTGCCAGCMGCCGCGGTAA-3′) and 907R (5′-CCGTCAATTCMTTTRAGTTT-3′). The internal transcribed spacer (ITS) region of fungal 18S rDNA gene was amplified with the primers ITS1F (5′-GGAAGTAAAAGTCGTAACAAGG-3′) and ITS2R (5′-GCTGCGTTCTTCATCGATGC-3′) under the following conditions: pre-denaturation at 98°C for 5 min, denaturation at 94°C for 30 s, annealing at 52°C for 30 s, extension at 72°C for 45 s and 30 cycles. The PCR products from different treatment samples were purified and pooled, and then sequenced on the Illumina Novaseq platform (Novaseq PE250, Illumina, CA, USA) by WEHEMO (Shenzhen, Guangdong, China). The raw sequences of different samples were separated using barcodes system, forward and reverse reads of the same sequence were combined using the FLASH tool. The QIIME DATA2 plugin was used for quality filtering, denoising, splicing and chimerism removal of the original data to get the clean data (Ma et al., 2021; Liu et al., 2022). The OTUs were classified using UCLUST software at 97% similarity level and the rarefaction analysis was conducted using the originally detected OTUs. The taxonomic assignment was conducted using the Ribosomal Database Project at minimal 80% confidence estimates (Ma et al., 2021; Song et al., 2023).

### 2.6 Functional gene abundance analysis

Using the extracted DNA as template, the relative expression abundances of microbial functional genes (*nifH*, *amoA-AOB*, *nirK* and *phoD*) involved in soil N and P cycling were detected by quantitative real-time PCR (qRT-PCR) which performed on the ABI StepOne instrument (ABI7500, Applied Biosystems, MA, USA) (Lang et al., 2020; Kim et al., 2022; Zhang Z. et al., 2022; Li et al., 2023). All the primer sequences were listed in Supplementary Table 1. The reaction system of qRT-PCR was 20 μL and included PCR reaction mixture contained 10 μL 2 × qPCR mix (2 × ChamQ Universal SYBR, Vazyme, Jiangsu, CHN), 0.8 μL primers (10 μM), 1.5 μL template DNA, 0.4 μL ROXII and 7.3 μL sterile ddH_2_O. The qRT-PCR procedure was as follows: pre-denaturation at 95°C for 5 min, denaturation at 95°C for 15 s, annealing at 55°C for 30 s, extension at 72°C for 30 s, 40 cycles, extension at 72°C for 10 min. For standard curve generation, the samples were amplified with primers and ligated to the Pgem-T Easy Vector after recycling the target DNA fragments, and then transformed into DH5a competent state. The standard curves were obtained by continuous dilutions of the known copy plasmid DNA inserted with the fragment, and the amplification efficiencies ranged from 87.29 to 93.09% for different genes.

### 2.7 Soil metabolite analysis

We tested the changes of metabolites in rhizosphere soil of *P. chinense* Schneid seedlings using untargeted metabolomics (WEHEMO, Shenzhen, Guangdong, China). Soil samples were individually grounded with liquid N and the homogenates were resuspended with prechilled 80% methanol (67-56-1, Thermo Fisher, CA, USA) by well vortex. The samples were incubated on ice for 5 min and then centrifuged at 15,000× *g*, 4°C for 20 min (D3024R, Scilogex, CT, USA). Some of supernatant was diluted to final concentration containing 53% methanol by LC–MS grade water (7732-12-5, Merck, Darmstadt, Germany). The samples were subsequently transferred to a new Eppendorf tube and centrifuged at 15,000× *g*, 4°C for 20 min (D3024R, Scilogex, CT, USA). An Orbitrap Q Exactive ™ HF mass spectrometer (Thermo Fisher, CA, USA) coupled with a Vanquish UHPLC system (Thermo Fisher, CA, USA) was used for an ultra-high performance liquid chromatography-tandem mass-spectrometry (UPLC-MS/MS) analysis (Want et al., 2013). Samples were injected onto a Hypesil Goldcolumn (100 mm × 2.1 mm, 1.9 μm) (Thermo Fisher, CA, USA) using a 17-min linear gradient at a flow rate of 0.2 mL min^−1^. The eluents for the positive polarity mode were eluent A (0.1% FA in Water) and eluent B (Methanol). The eluents for the negative polarity mode were eluent A (5 mM ammonium acetate, pH 9.0) and eluent B (Methanol).The solvent gradient was set as follows: 2% B, 1.5 min; 2–100% B, 12.0 min; 100% B, 14.0 min; 100–2% B, 14.1 min; 2% B, 17 min. Q ExactiveTM HF mass spectrometer was operated in positive/negative polarity mode with spray voltage of 3.5 kV, capillary temperature of 320°C, sheath gas flow rate of 35 psi and aux gas flow rate of 10 L min^−1^, S-lens RF level of 60, Aux gas heater temperature of 350°C (Cheng et al., 2022).

The raw data were processed using the compound discoverer 3.1 (CD3.1, Thermo Fisher, CA, USA) to perform peak alignment, peak picking and quantitation for each metabolite. We used the normalized data to predict molecular formulas based on additive ions, molecular ion peaks and fragment ions. Then we matched the peaks using the mzCloud, mzVault and MassList database to obtain the accurate quantitative results. We adopted the normalization function to make the data close to normal distribution, and applied a univariate analysis (*t*-test) to calculate the statistical significance (*p* < 0.05). The DAMs were screened based on log_2_(FC) > 1, *p* < 0.05, then enriched and annotated in the KEGG database.

### 2.8 Statistical analysis

The data were analyzed using SPSS software (V22.0, IBM, NY, USA) with a significant difference level. The normality of data was tested using the Shapiro–Wilk test and non-normal data was transformed with log_10_, square root or sine to fit a normal distribution. The significant differences of soil physicochemical properties, enzyme activities, gene expression levels and soil metabolites between N addition treatments were analyzed by the methods of one-way analysis of variance (ANOVA) and Kruskal-Wallis test. A nonmetric multidimensional scaling (NMDS) ordination to illustrate the clustering of bacterial and fungal community composition variation was conducted on the Bray-Curtis distance of the phylum and order. The variation between the groups in NMDS was tested with function *betadisper ()*, and rerified with a permutation test. The redundancy analysis (RDA) was chosen based on the first axis length in DCA analyses of bacteria and fungi (1.65 and 1.70 respectively). RDA were conducted on soil physicochemical properties with microbial diversities. Pearson correlation analysis and the T-test were performed to integrate and visualize the normalized and log-transformed datasets of sequencing and metabolomics. These graphs were conducted using R language (V3.5.1, Lucent, NJ, USA) and Origin software (2021 version, Origin Lab, MA, USA).

## 3 Results

### 3.1 Effects of N addition on soil physicochemical property and enzyme activity

Soil pH values significantly decreased by 15.75, 19.21 and 11.81%, respectively, in the N addition groups compared with CK, while they were no significant difference among N5, N10 and N15 treatment groups (Table 1). Under the N addition treatments, no significant changes were observed in the soil TN contents while the soil TP content significantly decreased in N10 treatment. In comparison with CK, the SOM content showed an evident increase under N5 treatment, but no notable difference was found in other two N treatment groups. Under the same condition, the soil AP content was evidently increased with 2.15-fold in N10 treatment group, while no significant changes were found in N5 and N15 treatments compared with CK (Table 1). The soil AK contents showed a distinct increase under N5 and N10 treatment groups, but there was no obvious change in N15 treatment group. Furthermore, the soil NH_4_^+^-N was increased with 9.5- and 3.01-fold, concurrently the soil NO_3_^−^-N was significantly increased with 1.97- and 0.86-fold in N10 and N15 treatment groups.

**Table 1:** Effects of N addition on soil physicochemical properties in rhizosphere soil of *P. chinense* Schneid seedlings.

Treatments	pH	SOM (g kg^−1^)	TN (g kg^−1^)	TP (g kg^−1^)	AP (mg kg^−1^)	AK (mg kg^−1^)	NH_4_^+^-N (mg kg^−1^)	NO_3_^−^-N (mg kg^−1^)
CK	6.35 ± 0.62 a	24.28 ± 0.52 b	1.74 ± 0.27 a	0.43 ± 0.05 a	49.79 ± 2.99 b	165.95 ± 8.73 b	6.73 ± 0.50 c	16.11 ± 1.26 c
N5	5.35 ± 0.23 b	29.25 ± 0.73 a	1.78 ± 0.11 a	0.40 ± 0.03 ab	59.00 ± 4.96 b	222.69 ± 35.65 a	8.00 ± 0.53 c	22.75 ± 2.57 c
N10	5.13 ± 0.10 b	23.49 ± 1.34 b	1.66 ± 0.18 a	0.34 ± 0.01 b	157.04 ± 20.65 a	199.34 ± 16.83 ab	70.67 ± 6.11 a	47.78 ± 4.76 a
N15	5.60 ± 0.04 b	25.14 ± 1.60 b	1.67 ± 0.08 a	0.43 ± 0.03 a	54.36 ± 4.87 b	168.67 ± 13.60 b	27.00 ± 3.27 b	30.00 ± 4.85 b

Data represent means ± SD, *n* = 3. The different letters following the numbers of the same parameter indicate significant difference (*p* < 0.05). SOM, soil organic matter; TN, total nitrogen; TP, total phosphorus; AP, available phosphorus; AK, available potassium. CK, control; N5, 5 g m^−2^; N10, 10 g m^−2^; N15, 15 g m^−2^.

The SU activities significantly increased by 59.55 and 21.15% in N5 and N10 treatment groups, but slightly decreased by 20.33% in the N15 group compared to CK (Table 2). Soil CAT activities significantly increased in N5 treatment compared to CK (*p* < 0.01). Conversely, soil UR activities were showed a notable reduction with 76.00, 30.00 and 60.00%, respectively, in N addition treatments.

**Table 2:** Effects of N addition on enzyme activities in rhizosphere soil of *P. chinense* Schneid seedlings.

Treatments	SU (mg g^−1^ d^−1^)	CAT (ml g^−1^ 20 min^−1^)	ACP (ug g^−1^ h^−1^)	UR (mg g^−1^ d^−1^)
CK	4.87 ± 0.11 bc	0.121 ± 0.01 b	25.29 ± 1.95 b	0.50 ± 0.09 a
N5	7.77 ± 1.24 a	0.188 ± 0.01 a	22.97 ± 1.25 b	0.12 ± 0.03 c
N10	5.90 ± 0.69 b	0.124 ± 0.02 b	48.51 ± 9.97 a	0.35 ± 0.03 b
N15	3.88 ± 0.72 c	0.129 ± 0.01 b	22.85 ± 5.32 b	0.20 ± 0.03 c

Data represent means ± SD, *n* = 3. The different letters following the numbers of the same parameter indicate significant difference (*p* < 0.05). SU, sucrase; CAT, catalase; ACP, acid phosphatase; UR, urease. CK, control; N5, 5 g m^−2^; N10, 10 g m^−2^; N15, 15 g m^−2^.

### 3.2 Effects of N addition on microbial diversity

**Figure 1:**
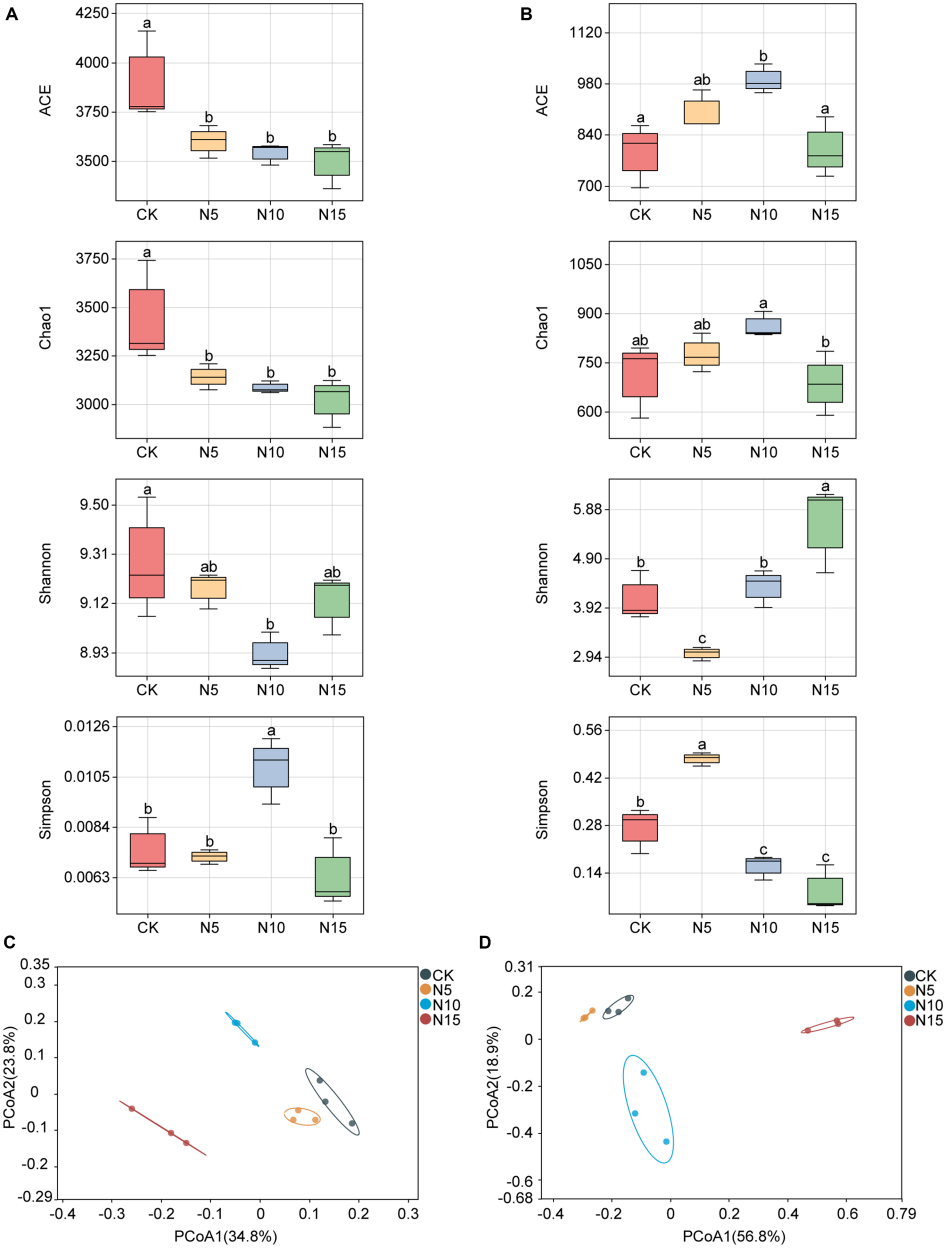
Effects of N addition on microbial diversity in rhizosphere soil of *P. chinense* Schneid seedlings. **(A)** Bacterial *α* diversity. **(B)** Fungal *α* diversity. **(C)** Bacterial *β* diversity. **(D)** Fungal *β* diversity. The different letters on the bar of the same parameter indicate significant difference (*p* < 0.05). CK, control; N5, 5 g m^−2^; N10, 10 g m^−2^; N15, 15 g m^−2^.

The ACE index of rhizosphere soil bacteria was significantly decreased, and both the indices of Chao1 and Shannon were notably reduced under N addition treatments compared with CK (Figure 1A). Meanwhile, the Simpson index of rhizosphere soil bacteria was evidently increased in N10 treatment, while no notable differences were observed in the N5 and N15 treatment samples compared with CK (Figure 1A).

The ACE index of rhizosphere soil fungi exhibited a significant increase under the N5 and N10 treatments, but showed no significant difference in the N15 treatment group compared to the control (CK) (Figure 1B). The Chao1 index showed a notable increase in the N10 treatment but a reduction in the N15 treatment. At the same time, the Shannon indices increased in both the N10 and N15 treatments but decreased in the N5 treatment compared to the CK. Furthermore, the Simpson index of rhizosphere soil fungi showed a substantial increase under the N5 treatment, while it significantly decreased in both the N10 and N15 treatment groups in comparison to the CK (Figure 1B). The PCoA analysis revealed noticeable clustering of bacterial and fungal communities in the N5 and CK treatment samples, while they appeared distinctly separated with the N10 and N15 treatments (Figures 1C,D).

### 3.3 Effects of N addition on microbial community

**Figure 2:**
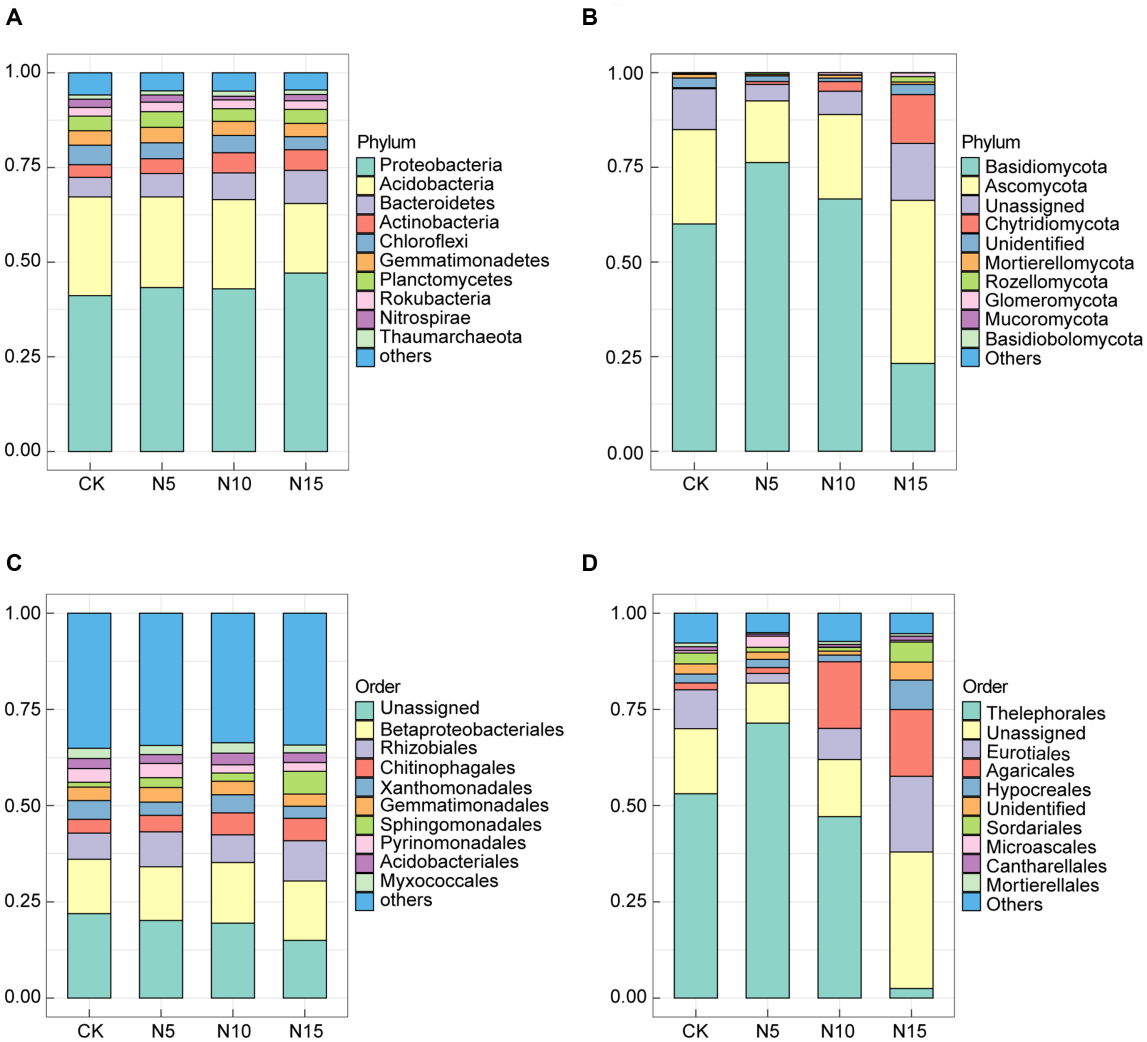
Effects of N addition treatments on microbial community composition in rhizosphere soil of *P. chinense* Schneid seedlings. **(A)** Bacterial phylum. **(B)** Fungal phylum. **(C)** Bacterial order. **(D)** Fungal order. CK, control; N5, 5 g m^−2^; N10, 10 g m^−2^; N15, 15 g m^−2^.

Under N addition treatments, the *Proteobacteria* (43.13% ~ 47.11%), *Acidobacteria* (18.40% ~ 26.10%), *Bacteroidetes* (5.18% ~ 8.73%), *Actinobacteria* (3.32% ~ 5.45%), *Chloroflexi* (3.43% ~ 5.16%), *Gemmatimonadetes* (3.54% ~ 4.06%), *Planctomycetes* (3.34% ~ 4.15%), *Rokubacteria* (2.29% ~ 2.49%), *Nitrospirae* (0.98% ~ 2.18%) and *Thaumarchaeota* (1.10% ~ 1.37%) were the top 10 bacterial phyla in terms of the relative abundance. Notably, the *Proteobacteria*, *Acidobacteria* and *Bacteroidetes* emerged as the dominant bacteria phyla, collectively constituting 72.40% ~ 74.23% of the total bacterial population (Figure 2A). Comparatively, the relative abundances of *Proteobacteria*, *Bacteroidete*s and *Actinobacteria* increased, while the relative abundances of *Acidobacteria* and *Chloroflexi* decreased in the N application groups when compared to CK (Supplementary Table 2). At order level, the *Betaproteobacteriales* (13.98% ~ 15.75%), *Rhizobiales* (6.79% ~ 10.43%), *Chitinophagales* (3.60% ~ 5.87%), *Xanthomonadales* (3.13% ~ 4.90%), *Gemmatimonadales* (3.17% ~ 3.83%), *Sphingomonadales* (1.25% ~ 5.94%), *Pyrinomonadales* (2.17% ~ 3.70%), *Acidobacteriales* (2.32% ~ 3.04%) and *Myxococcales* (2.00% ~ 2.69%) were the top 9 bacterial order in terms of the relative abundance, among the *Betaproteobacteriales*, *Rhizobiales* and *Chitinophagales* were the predominant bacteria which accounting for 24.58 ~ 31.76% in bacterial communities under N treatments (Figure 2C).

In the fungal communities, the *Basidiomycota* (21.80% ~ 76.65%), *Ascomycota* (15.98% ~ 43.42%), Unassigned (4.32% ~ 15.06%), *Chytridiomycota* (0.28% ~ 13.44%), Unidentified (0.90% ~ 2.70%), *Mortierellomycota* (0.37% ~ 1.07%), *Rozellomycota* (0.12% ~ 1.51%), *Glomeromycota* (0.08% ~ 1.04%), *Mucoromycota* (0.01% ~ 0.04%) and *Basidiobolomycota* (0.00% ~ 0.06%) were the top 10 fungi phyla in terms of the relative abundance, among the *Basidiomycota*, *Ascomycota* and *Chytridiomycota* emerged as dominant fungi which consisting 78.66 ~ 93.22% of total fungi (Figure 2B). Notably, the relative abundances of *Basidiomycota* and *Chytridiomycota* increased in the N10 treatment group, while the relative abundances of *Ascomycota, Mortierellomycota* and *Rozellomycota* decreased in N application groups compared to CK (Supplementary Table 3). At order level, the *Thelephorales* (2.42% ~ 71.58%), Unassigned (10.40% ~ 35.49%), *Eurotiales* (2.44% ~ 19.84%), *Agaricales* (1.56% ~ 17.69%), *Hypocreales* (1.82% ~ 7.93%), Unidentified (1.05% ~ 4.67%), *Sordariales* (0.94% ~ 4.98%), *Microascales* (0.09% ~ 2.47%), *Cantharellales* (0.52% ~ 1.18%) and *Mortierellales* (0.37% ~ 1.07%) in the rhizosphere soil were the top 10 fungi order in the light of the relative abundance, among the *Thelephorales*, *Eurotiales* and *Agaricales* were the primary fungi which accounting for 38.13 ~ 75.58% in fungal communities under N input treatments (Figure 2D).

### 3.4 Effects of N addition on gene expression abundance

**Figure 3:**
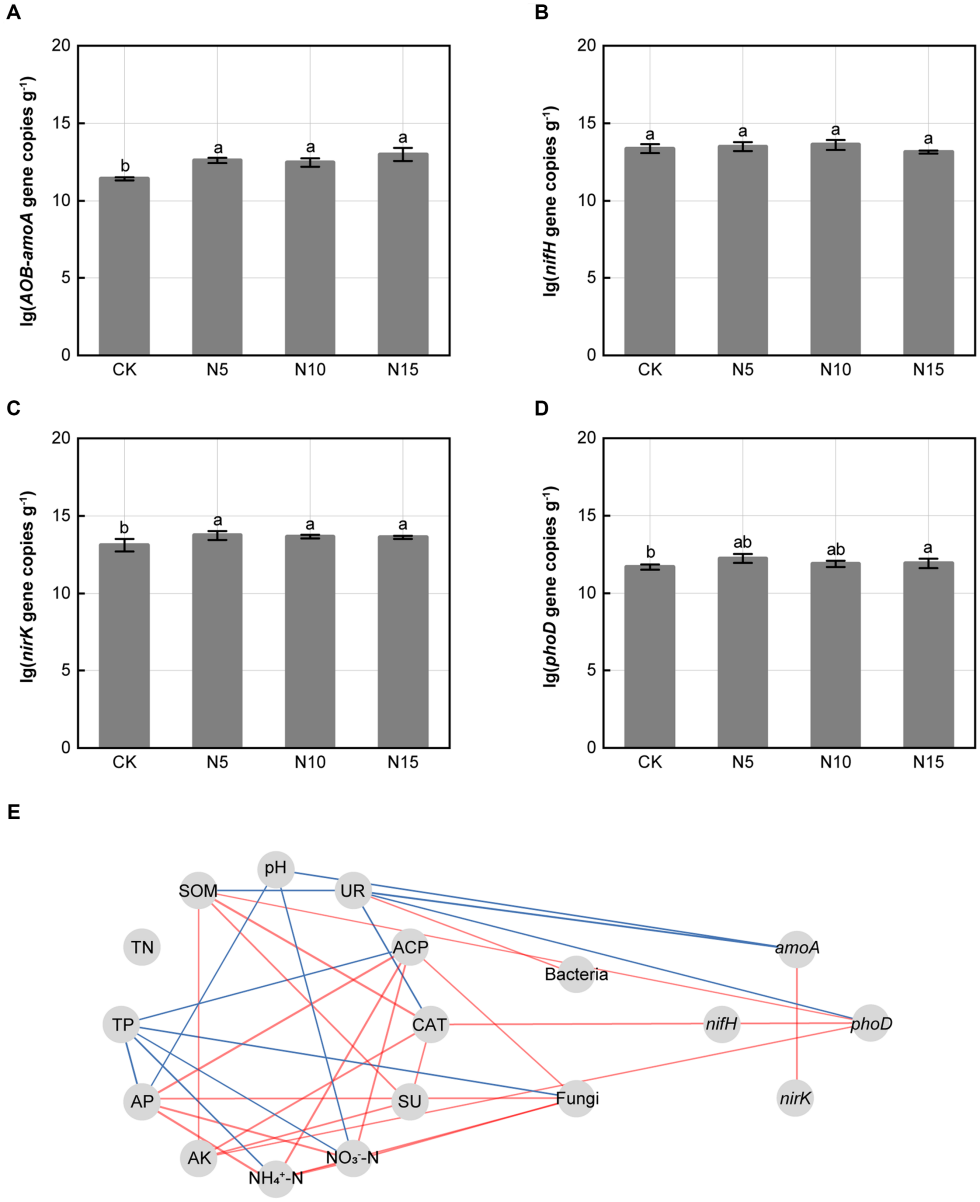
Effects of N addition treatments on the relative expression abundances of functional genes and correlation analysis. **(A)** *AOB-amoA* gene, **(B)** *nifH* gene, **(C)** *nirK* gene, **(D)** *phoD* gene. **(E)** Correlation analysis of soil physicochemical properties, enzyme activities, functional genes and microbial diversity. SOM, soil organic matter; TN, total nitrogen; TP, total phosphorus; AP, available phosphorus; AK, available potassium; SU, sucrase; CAT, catalase; ACP, acid phosphatase; UR, urease. The different letters on the bar of the same parameter indicate significant difference (*p* < 0.05). The lines marked with red and blue color represent the significant positive and negative correlations, respectively, (*p* < 0.05). CK, control; N5, 5 g m^−2^; N10, 10 g m^−2^; N15, 15 g m^−2^.

Under N addition treatments, the relative expression abundances of *amoA*, *nirK* and *phoD* genes related to N and P metabolism in microbe significantly increased when compared with CK (Figures 3A,C,D). However, the relative expression abundances of *nifH* gene in microbe were no notable difference in comparison with CK (Figure 3B). The *amoA* gene was negatively correlated with soil UR and soil pH. The *phoD* gene was positively correlated with soil CAT, soil SOM and AK, but negatively correlated with soil UR (*p* < 0.05). Furthermore, *amoA* gene presented a significant positive correlation with *nirK* gene (*p* < 0.01) (Figure 3E).

### 3.5 Correlation and redundancy analysis

**Figure 4:**
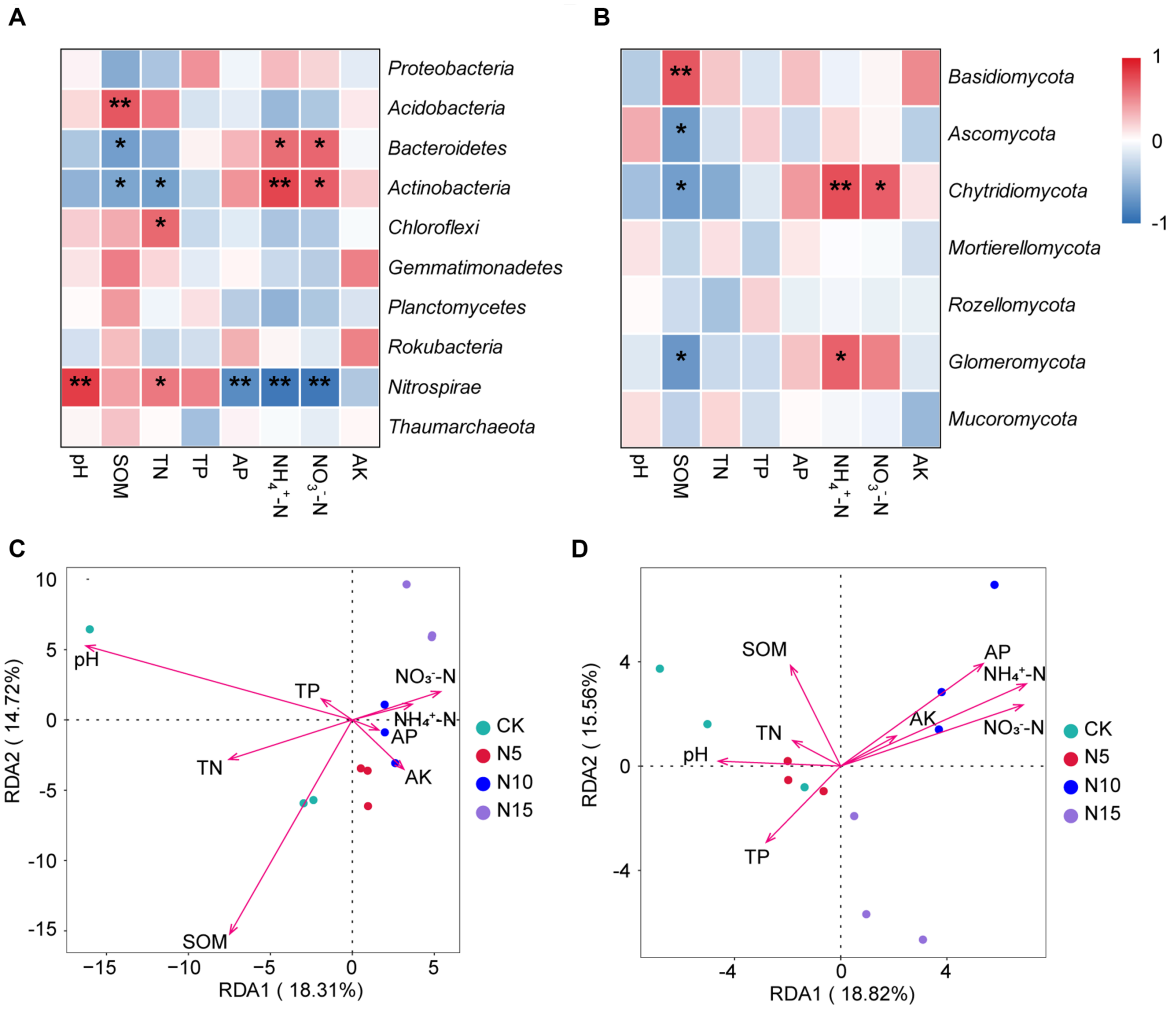
Correlation and RDA analyses of dominant microorganisms at phylum level and microbial communities with soil physicochemical properties under N addition treatments. **(A)** The heatmap of correlation between Top 10 bacteria and soil properties. **(B)** The heatmap of correlation between Top 7 fungi and soil properties. **(C)** RDA analysis between bacterial community and soil physicochemical property. **(D)** RDA analysis between fungal community and soil physicochemical property. SOM, soil organic matter; TN, total nitrogen; TP, total phosphorus; AP, available phosphorus; AK, available potassium. Significant levels: ** indicates *p* < 0.01, * indicates *p* < 0.05. CK, control; N5, 5 g m^−2^; N10, 10 g m^−2^; N15, 15 g m^−2^.

The *Nitrospirae* was negatively correlated with soil NH_4_^+^-N, NO_3_^−^-N and AP, but positively correlated with soil pH and TN (*p* < 0.05). The *Chloraflexi* was positively correlated with soil TN (*p* < 0.05). The *Actinobacteria* exhibited a significant positive correlation with soil NH_4_^+^-N and NO_3_^−^-N (*p* < 0.01), but displayed a notable negative correlation with soil TN and SOM (*p* < 0.05). The *Bacteroidetes* showed a substantial positive correlation with soil NH_4_^+^-N and NO_3_^−^-N, but while exhibiting a significant negative correlation with soil SOM (*p* < 0.05). Furthermore, the *Acidobacteria* showed a obvious positive correlation with soil SOM (*p* < 0.01) (Figure 4A). Based on RDA analysis, the first two principal components between bacterial community and physicochemical properties explained 18.31 and 14.72%. Among these, the soil pH (*r*^2^ = 0.7916, *p* = 0.0030) and SOM (*r*^2^ = 0.7972, *p* = 0.0015) emerged as the dominant regulating factors in bacterial community composition (Figure 4C; Table 3).

**Table 3:** Monte Carlo permutation test for influencing factors of microbial community structure in rhizosphere soil of *P. chinense* Schneid seedlings under N addition.

Factors	Community structure			
	Bacteria		Fungi	
	*r* ^2^	*p*	*r* ^2^	*p*
pH	0.7972	0.0030	0.5384	0.0410
SOM	0.7918	0.0015	0.5015	0.0390
TN	0.3774	0.1109	0.2438	0.2814
TP	0.1166	0.5907	0.4734	0.0485
AP	0.0869	0.6617	0.7726	0.0060
NH_4_^+^-N	0.1821	0.4363	0.8899	0.0010
NO_3_^−^ -N	0.2714	0.2454	0.8423	0.0005
AK	0.2234	0.3028	0.2819	0.2429

SOM, soil organic matter; TN, total nitrogen; TP, total phosphorus; AP, available phosphorus; AK, available potassium.

The *Glomeromycota* showed a significant positive correlation with soil NH_4_^+^-N, but presented a significant negative correlation with soil SOM (*p* < 0.05). The *Chytridiomycota* showed a substantial positive correlation with soil NH_4_^+^-N and NO_3_^−^-N, but had a notable negative correlation with soil SOM (*p* < 0.05). The *Basidiomycete* was significant positively correlated with soil SOM (*p* < 0.01), while the *Ascomycota* had a obvious negative correlation with soil SOM (*p* < 0.05) (Figure 4B). Additionally, The RDA analysis results showed that the first two principle components of fungal community explained 18.82 and 15.56%, among which the soil pH (*r*^2^ = 0.5384, *p* = 0.0410), SOM (*r*^2^ = 0.5015, *p* = 0.0390), TP (*r*^2^ = 0.4734, *p* = 0.0485) NH_4_^+^-N (*r*^2^ = 0.8899, *p* = 0.0010), NO_3_^−^-N (*r*^2^ = 0.8423, *p* = 0.0005) and AP (*r*^2^ = 0.7726, *p* = 0.0060) were the primary regulating factors for fungal community components (Figure 4D; Table 3).

### 3.6 Effects of N addition on rhizosphere soil metabolite

**Figure 5:**
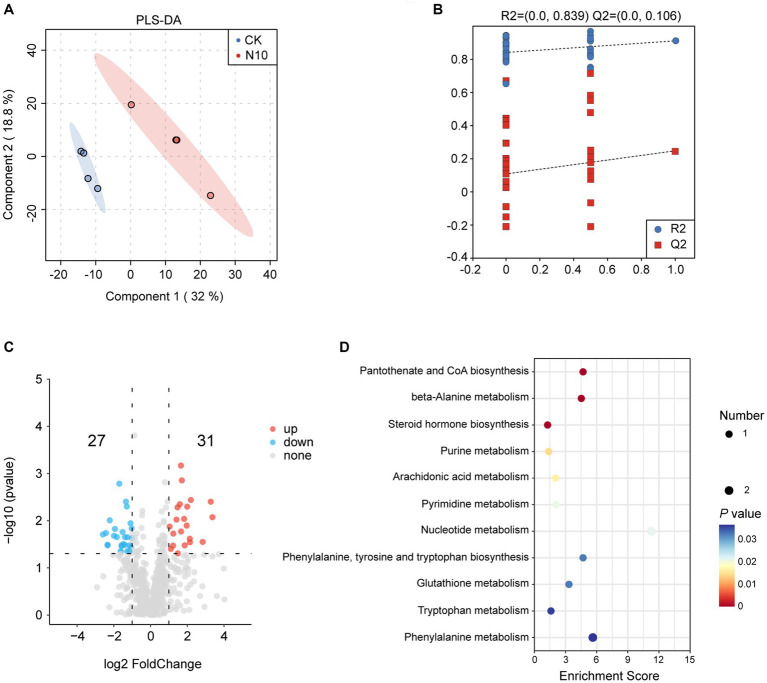
Effects of N addition on metabolites in rhizosphere soil of *P. chinense* Schneid seedlings. **(A)** PLS-DA analysis of metabolism between CK and N10. **(B)** Cross-validation model of PLS-DA. **(C)** The expression volcano map of DAMs. **(D)** The enrichment analysis of 7 DAMs in KEGG database. CK, control; N5, 5 g m^−2^; N10, 10 g m^−2^; N15, 15 g m^−2^.

To further assess the influence of N addition on the microenvironment in rhizosphere soil of *P. chinense* Schneid seedlings, we performed a metabolomics analysis of both CK and N10 samples using the UPLC-MS/MS platform. The PLS-DA analysis showed that the metabolites were obviously separated under CK and N10 treatments, indicating that N10 treatment exerted a significant effect on rhizosphere soil metabolites (Figure 5A). The cross-validation model of PLS-DA implied that the model was reliable and suitable for the screening of DAMs (R2Y = 0.839, Q2Y = 0.106) (Figure 5B). The volcano map analysis showed that a total of 58 DAMs (31 upregulated and 27 downregulated) were identified between CK and N10 treatment samples (log_2_(FC) > 1, *p* < 0.05) (Figure 5C). Notably, seven DAMs, including D-phenylalanine, phenylacetylglycine, uracil, indole, *γ*-glutamylalanine, prostaglandin F2α and guanine were enriched in various metabolic pathways, such as pantothenate and CoA biosynthesis, *β*-alanine metabolism, steroid hormone biosynthesis, purine metabolism, arachidonic acid metabolism, pyrimidine metabolism, nucleotide metabolism, phenylalanine tyrosine and tryptophan biosynthesis, glutathione metabolism, tryptophan metabolism and phenylalanine metabolism, as identified in the KEGG database (Figure 5D). Furthermore, under the N10 treatment, the absolute abundance of D-phenylalanine and phenylacetylglycine significantly decreased, while the absolute abundance of uracil, indole, *γ*-glutamylalanine, prostaglandin F2α and guanine distinctly increased (Supplementary Figure 1).

**Figure 6:**
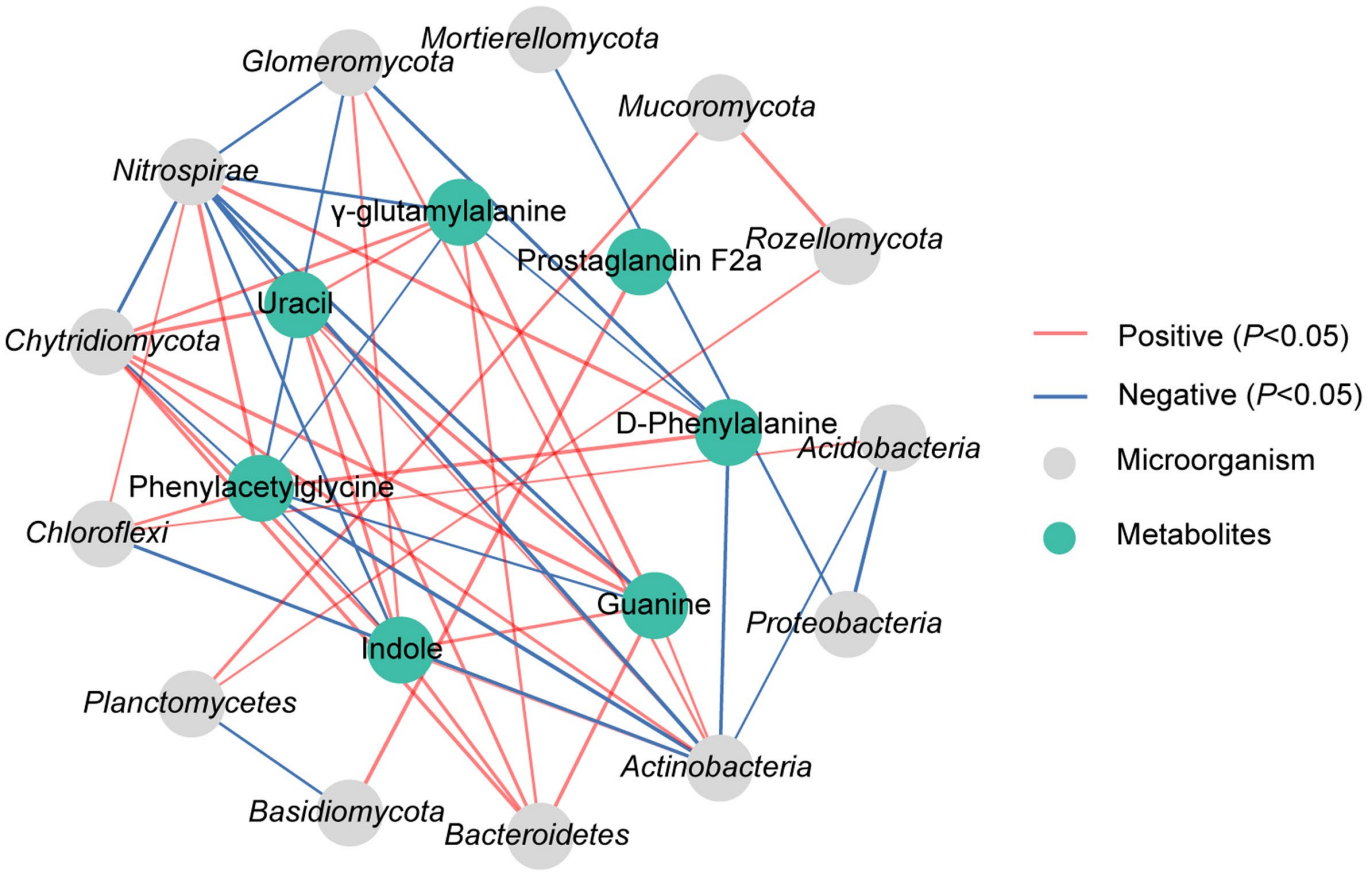
Correlation analysis of dominant microorganisms at phylum with DAMs in rhizosphere soil under N10 treatment. The lines marked with red and blue colors represent the significant positive and negative correlations, respectively, (*p* < 0.05). Only the indicators that have a significant correlation are shown in the figure.

The correlation analysis revealed significant associations between DAMs and dominant microorganisms, which the D-phenylalanine presented a significant positive correlation with *Nitrospirae* but a notable negative correlation with *Actinobacteria* and *Glomeromycota* (*p* < 0.05). Phenylacetylglycine showed a distinct positive correlation with *Chloroflexi* and *Nitrospirae*, while displaying a notable negative correlation with *Actinobacteria*, *Chytridiomycota* and *Glomeromycota* (*p* < 0.05). Uracil showed a significant positive correlation with *Bacteroidetes*, *Actinobacteria* and *Chytridiomycota*, while having an obvious negative correlation with *Nitrospirae* (*p* < 0.05). Indole exhibited a significant positive correlation with *Bacteroidetes*, *Actinobacteria*, *Chytridiomycota* and *Glomeromycota*, and a notable negative correlation with *Nitrospirae* (*p* < 0.05). It was also found that *γ*-glutamylalanine demonstrated a substantial positive correlation with *Bacteroidetes* and *Chytridiomycota*, while showing an obvious negative correlation with *Nitrospirae* (*p* < 0.05). Prostaglandin F2α displayed a significant positive correlation with *Basidiomycota* (*p* < 0.05). Guanine showed a notable positive correlation with *Bacteroidetes*, *Actinobacteria* and *Chytridiomycota*, while presenting a significant negative correlation with *Nitrospirae* (*p* < 0.05) (Figure 6).

The correlation analysis revealed significant associations between DAMs and soil physicochemical properties, which the D-phenylalanine presented a significant positive correlation with soil pH, TP and UR, but a notable negative correlation with AP, NH_4_^+^-N, NO_3_^−^-N, AK and ACP (*p* < 0.05). Phenylacetylglycine showed a significant positive correlation with soil pH, while having an obvious negative correlation with AP, NH_4_^+^-N, NO_3_^−^-N, AK and ACP (*p* < 0.05). The uracil exhibited a significant positive correlation with soil AP and SU, and an evident negative correlation with UR (*p* < 0.05). It was also found that indole demonstrated a substantial positive correlation with soil AP, NH_4_^+^-N, SU and ACP (*p* < 0.05). The *γ*-glutamylalanine displayed a significant positive correlation with AP, NH_4_^+^-N and NO_3_^−^-N (*p* < 0.05). Guanine showed a notable positive correlation with AP, NH_4_^+^-N, NO_3_^−^-N and SU (*p* < 0.05) (Supplementary Figure 2).

## 4 Discussion

### 4.1 N addition altered the rhizosphere soil physicochemical property and enzyme activity

N is a crucial element in soil, with substantial effects on physicochemical properties, enzyme activities, and the relative expression abundances of genes related to nutrient element metabolism (Cardinale et al., 2020; Chen et al., 2021; Zhang H. et al., 2022). Previous studies showed that N addition significantly decreased the soil pH value and SOC content, but increased the contents of soil NO_3_^−^-N, TN and available N in soil of wheat and *Cunninghamia lanceolata* forests, suggesting that N input regulated rhizosphere soil physicochemical properties by promoting soil acidification (Liu et al., 2022; Xu et al., 2022). In our present study, N addition led to a noticeable decrease in soil pH while increasing soil NH_4_^+^-N and NO_3_^−^-N levels, suggesting that N addition also promoted soil acidification and the accumulation of available N in the rhizosphere soil of *P. chinense* Schneid seedlings. The increase in soil NH_4_^+^-N and NO_3_^−^-N contents could be attributed to the upregulation of relative expression abundances of *amoA* and *nirK* genes, which are involved in nitrification processes. Additionally, our results indicated a significant positive correlation between soil available N and fungal diversity (Supplementary Figure 2), suggesting that the presence of available N supports fungal proliferation. Concurrently, the protons are generated in nitrification processes or secretes from the root following NH_4_^+^-N uptake, contributing to soil acidification (Hao et al., 2020). These findings collectively demonstrated that N addition promoted soil acidification and the accumulation of available N in the rhizosphere soil of *P. chinense* Schneid seedlings.

N input has no effect on the contents of the TN and TOC, but it enhanced the activities of urease and acid phosphatase in soil of bamboo forests (Tu et al., 2014). In our study, the soil TN contents did not exhibit significant changes, but the UR activities were notably reduced after N application. In the early stage after N input, soil N fertilizer was rapidly degraded into available N and were subsequently utilized by both roots and microorganisms. This dynamic process contributed to the maintenance of stable TN levels in the soil. The UR activities were reduced due to the decrease of catalytic substrate during the late period of N application (Xu et al., 2022). Under N10 treatment, the soil TP content was evidently reduced, whereas significantly increased the AP and AK contents, ACP activity and the relative expression abundances of *amoA* and *phoD* genes, indicating that appropriate N application could promote the transformation and accumulation of AP in soil through improving ACP activity and gene expression abundance, combining with available N to promote root development of *P. chinense* Schneid seedlings (data not shown). Furthermore, N input regulated the activities of SU and CAT, implying that they were involved in decomposition of the SOM and hydrogen peroxide in soil of the seedlings.

### 4.2 N addition regulated microbial diversity and community composition

N input influences the diversity and community composition of microorganism in rhizosphere soils of plants (Cardinale et al., 2020; Liu et al., 2021; Ma et al., 2021; Si et al., 2022). Under long-term N addition, the soil bacterial diversity decreased in agro-ecosystems, which was positively related to both soil pH and SOC, but increased the relative abundances of *Proteobacteria* and *Actinobacteria* (Dai et al., 2018). In this study, it was found that N addition significantly reduced the indices of Chao1 and Shannon, and produced obvious separation effect of bacterial communities in rhizosphere soil under N10 and N15 treatments compared with CK and N5 experimental groups, indicating that N input reduced bacterial diversity in rhizosphere soil of *P. chinense* Schneid seedlings. Under the same condition, the bacterial relative abundance in the rhizosphere soil were changed. At the bacterial phyla level, the *Proteobacteria*, *Acidobacteria* and *Bacteroidetes* were dominant bacteria phyla. The N addition directly and indirectly increased the soil nutrient content, and it could be benefit to the proliferation of eutrophic bacteria, which likely contributed to the increased relative abundance of eutrophic bacteria (*Proteobacteria*, *Acidobacteria* and *Bacteroidetes*) and made them the main bacteria phyla (Chen et al., 2023). There were noticeable increases in the relative abundances of *Proteobacteria*, *Bacteroidetes* and *Actinobacteria* with increasing N addition. Conversely, there were decreases in the relative abundances of *Acidobacteria* and *Chloroflexi*, suggesting that N addition could promote the eutrophic rather than oligotrophic and shift bacterial communities to more eutrophic taxa (Liu et al., 2020, 2023). The *Bacteroidetes* and *Actinobacteria* showed a significant negative position with SOM, suggesting that N addition could promote the carbon utilization efficiency by increasing the relative abundance of eutrophic bacteria in rhizosphere soil of *P. chinense* Schneid seedlings (Liu et al., 2020). Importantly, despite these shifts in relative abundances, the overall composition of the bacterial community remained relatively stable under the N addition treatments, suggesting that N input had the effect of the relative abundance of specific bacterial phyla while little influencing the bacterial community in rhizosphere soil of *P. chinense* Schneid seedlings. Recent studies have shown that nutrients availability was a predictor of bacteria diversity in tropical forest ecosystem, implying that N input directly or indirectly regulated the bacterial diversity and the relative abundance of main bacterial phyla by modulating physicochemical properties (Cui et al., 2021). In the present experiment, N application enhanced the soil available N content, suggesting that N addition decreased bacterial diversity through improving nutrient availability in rhizosphere soil of *P. chinense* Schneid seedlings. Meanwhile, N input reduced soil pH and regulated SOM content, which had significant correlation with the relative abundances of main bacterial phyla, implying that N addition indirectly affected the relative abundances of domain bacterial phyla by regulating physicochemical properties in rhizosphere soil of *P. chinense* Schneid seedlings (Du et al., 2019; Li et al., 2019). Additionally, the berberine synthesized in root were secreted into soil, which might inhibit the growth and proliferation of key bacterial phyla in soil, and then reduced its bacterial diversity and relative abundance.

In the subtropical forests, the Shannon and Chao1 indices of fungus in rhizosphere soil were significantly increased, and also enhanced the relative abundances of *Mortierellomycota* and *Rozellomycota* under appropriate N input, which had distinctly correlation with the soil NH_4_^+^-N and NO_3_^−^-N, indicating that N input elevated the diversity and relative abundance of soil fungi in subtropical forests by regulating soil physicochemical properties (Wang J. et al., 2021). In the present study, N input increased the fungal ACE index, and appropriate N addition distinctly elevated the fungal indices of Chao1, Shannon and Simpson, which presented a significant positive correlation with soil AP, NH_4_^+^-N and NO_3_^−^-N (*p* < 0.05) (Supplementary Figure 2), suggesting that appropriate N input might significantly enhances the fungal diversity through promoting the N metabolism in rhizosphere soil of *P. chinense* Schneid seedlings. Our results were in agreement with the findings of previous studies in the subtropical forests (He et al., 2021; Wang J. et al., 2021). Under the same condition, the fungal community compositions were not obviously changed, but the relative abundance of *Basidiomycota* and *Chytridiomycota* were enhanced under the N10 application, which the *Basidiomycota* was eutrophic taxa and *Chytridiomycota* showed a significant correlation with soil NH_4_^+^-N and NO_3_^−^-N (*p* < 0.05), implying that N input elevated the relative abundances of *Basidiomycota* and *Chytridiomycota* by promoting the N metabolism in soil, because the soil available N provides available nutrients for the growths and proliferations of *Basidiomycota* and *Chytridiomycota* (Digby et al., 2010; Liu et al., 2020). At the same time, N addition reduced the relative abundance of *Mortierellomycota*, but enhanced the relative abundances of other domain fungal phyla in appropriate N treatment groups, suggesting that different fungal groups adopt various mechanisms to response N fertilizer applications. These results were consistent with the results of RDA analysis.

### 4.3 N addition regulated soil metabolism

Soil metabolites are mainly derived from microbial metabolism, which change reveals the microbial response to soil nutrients, and they are affected by N addition (Cheng et al., 2022). In this study, a total of 7 DAMs were identified in rhizosphere soil under N10 treatment, they were primarily involved in the metabolism pathways of nucleotide, phenylalanine and hormone. N10 application significantly increased the absolute abundances of guanine and uracil that an important components of nucleotides, which had a significant positive correlation with soil NH_4_^+^-N, NO_3_^−^-N, AP and SU (Supplementary Figure 2), suggesting that N10 addition promoted nucleotide synthesis by improving the capacity of soil N metabolism in rhizosphere soil of *P. chinense* Schneid seedlings (Tang et al., 2022). At the same time, guanine and uracil presented a significant positive correlation with *Bacteroidetes*, *Actinobacteria* and *Chytridiomycota*, proposing that N10 addition promoted nucleic acid synthesis by improving the relative abundances of these three fungi.

Indeed, the *γ*-glutamylalanine is the precursor of L-glutamic acid that related to the N response and urea cycle (Wang et al., 2020; Lian et al., 2021; Navarro-León et al., 2022; Wang et al., 2023). N10 application significantly increased the absolute abundance of *γ*-glutamylalanine in rhizosphere soil which showed a significant positive correlation with soil NH_4_^+^-N, NO_3_^−^-N and *amoA* gene, suggesting that N10 addition promoted the transformation and utilization of N nutrient through improving the capacity of amino acid metabolism in rhizosphere soil of *P. chinense* Shneid seedlings (Wang et al., 2020; Cheng et al., 2022; Wang et al., 2023). Meanwhile, the *γ*-glutamylalanine exhibited a significant positive correlation with *Bacteroidetes* and *Chytridiomycota*, suggesting that N10 addition improved the *γ*-glutamylalanine synthesis by promoting microbial proliferation. Furthermore, N10 input evidently enhanced the absolute abundance of indole and prostaglandin F2α. The former is synthesized by *Proteobacteria* and is the precursor of auxin, the latter regulates the lipid metabolism and Ca^2+^ signal pathway, these results are consistent with the relative abundance of dominant bacterial phyla and fungal phyla in rhizosphere soil of *P. chinense* Schneid seedlings.

Under the same condition, N10 input evidently reduced the absolute abundance of D-phenylalanine, and had a significant positive correlation with *Nitrospirae*, suggesting that N10 addition decreased the D-phenylalanine content through inhibited the *Nitrospirae* proliferation in rhizosphere soil. The soil phenylalanine is absorbed and utilized by plant root for synthesizing lignin and flavonoids, and promotes root growth and secondary metabolism, while leads to reduce its content, subsequently decreases the nitration and loss of N element in soil (Li et al., 2017; Fu et al., 2023). Furthermore, N10 application decreased the absolute abundance of soil phenylacetylglycine which showed a significant correlation with *Nitrospirae*, *Chloroflexi*, *Actinobacteria*, *Chytridiomycota* and *Glomeromycota*, proposing that N10 addition reduced the phenylacetylglycine content by inhibiting some microbial proliferation and metabolism in rhizosphere soil of *P. chinense* Schneid seedlings. The phenylacetylglycine is used to synthesize antibiotics, while promotes or represses other microbial growth, ultimately improves the efficiency of N utilization of *P. chinense* Schneid seedlings (Fu et al., 2023).

**Figure 7:**
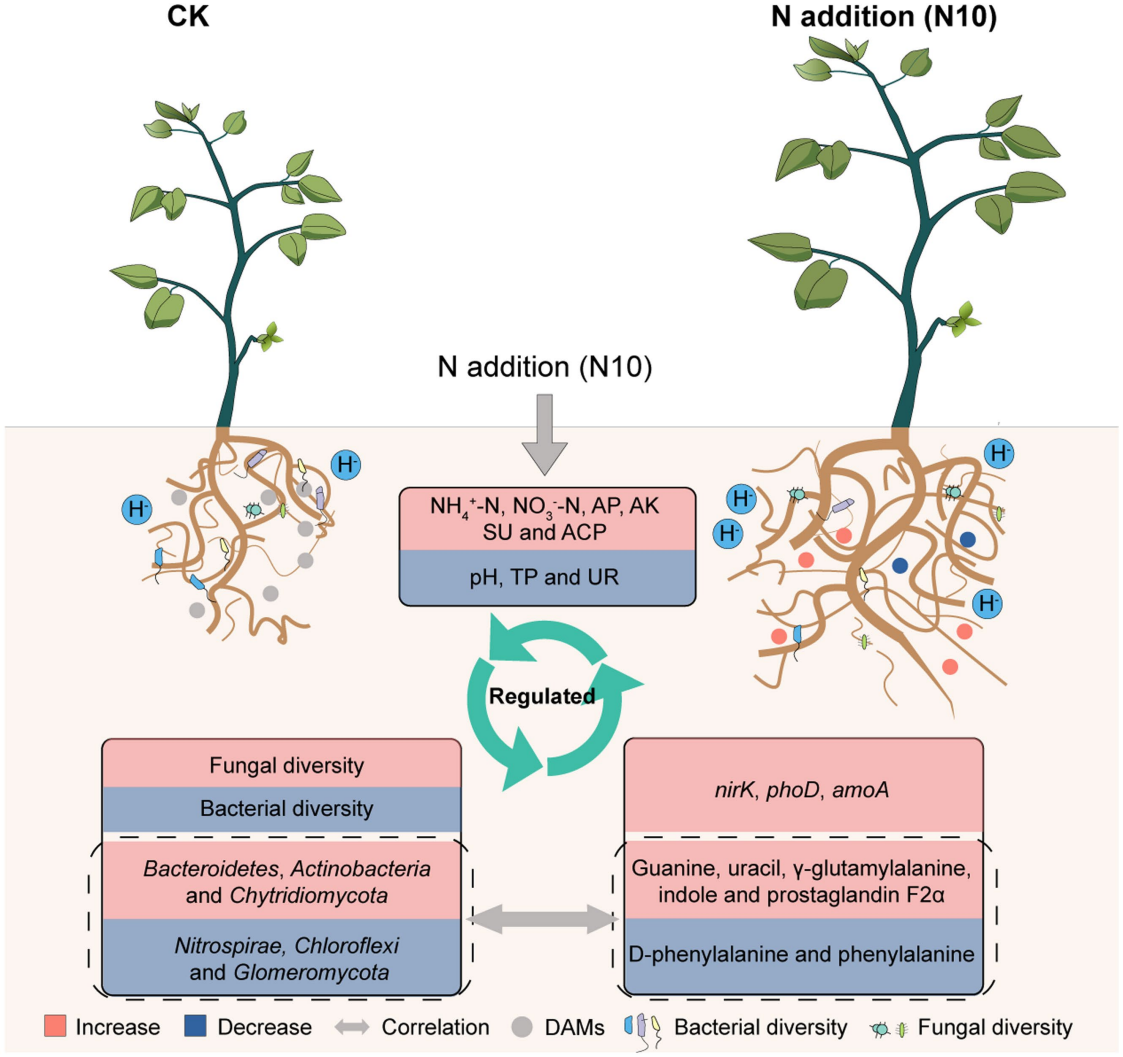
Theoretical mechanism of microorganisms and metabolic function reponse to N addition in rhizosphere soil of *P. chinense* Schneid seedlings. TP, total phosphorus; AP, available phosphorus; AK, available potassium; ACP, acid phosphatase; UR, urease; CK, control; N5, 5 g m^−2^; N10, 10 g m^−2^; N15, 15 g m^−2^.

Based on our results, a conceptual model was developed for describing the response mechanism of microorganisms and metabolic function to N addition in rhizosphere soil of *P. chinense* Schneid seedlings. The appropriate N addition (N10) decreased the soil pH, TP and UR activity, concurrently enhanced the available nutrient content, SU activity and relative expression abundances of *amoA*, *nirK* and *phoD* genes, subsequently elevated the fungal diversity and the relative abundances of *Bacteroidetes*, *Actinobacteria* and *Chytridiomycota*, simultaneously reduced the bacterial diversity and the relative abundances of *Nitrospirae*, *Chloroflexi* and *Glomeromycota*, ultimately increased the absolute contents of uracil, indole, *γ*-glutamylalanine, prostaglandin F2α and guanine while reduced the levels of D-phenylalanine and phenylacetylglycine, suggesting that N10 addition regulated the microbial community abundance and metabolic function through improving nutrient cycle in rhizosphere soil of *P. chinense* Schneid seedlings (Figure 7).

## 5 Conclusion

In summary, N addition significantly increased the contents of AP, AK, NH_4_^+^-N, NO_3_^−^-N, SU activity, and the relative expression abundances of *amoA* and *phoD* genes, concurrently decreased soil pH, TP content and UR activity, demonstrating that N addition promoted acidification and nutrient cycle in rhizosphere. Meanwhile, N addition enhanced the fungal diversity and the relative abundances of *Bacteroidetes*, *Actinobacteria* and *Chytridiomycota*, reduced the bacterial diversity and the relative abundances of *Nitrospirae*, *Chloroflexi* and *Glomeromycota*, which exhibited a significant correlation with soil NH_4_^+^-N, NO_3_^−^-N, AP and UR, indicating that N addition regulated the microbial diversity and community abundance through modulating the physicochemical properties. Additionally, N10 addition distinctly improved the absolute abundances of the uracil, guanine, indole, prostaglandin F2α and *γ*-glutamylalanine, whereas reduced the absolute contents of D-phenylalanine and phenylacetylglycine, which showed a significant correlation with soil NH_4_^+^-N, NO_3_^−^-N, AP, *Bacteroidetes*, *Chytridiomycota*, *Actinobacteria* and *Nitrospirae*, suggesting that N10 addition impacted the metabolic function through regulating nutrient cycle and microbial community abundance. Therefore, our results indicates that N10 addition regulates microbial community abundance and metabolic function by enhancing nutrient cycling capacity in the rhizosphere soil of *P. chinense* Schneid seedlings.

## Data availability statement

The datasets presented in this study can be found in online repositories. The names of the repository/repositories and accession number(s) can be found at: NCBI – PRJNA1035961.

## Author contributions

YG: Writing – original draft, Data curation, Formal analysis, Investigation, Software, Visualization. XLC: Data curation, Formal analysis, Writing – original draft. YS: Writing – review & editing. XYC: Supervision, Validation, Writing – review & editing. GH: Conceptualization, Funding acquisition, Writing – review & editing. XH: Funding acquisition, Writing – review & editing. GW: Funding acquisition, Writing – review & editing. HH: Conceptualization, Funding acquisition, Investigation, Methodology, Writing – review & editing. ZL: Writing – review & editing, Conceptualization, Investigation, Methodology, Supervision.

## References

[ref1] Cardinale M. Ratering S. Sadeghi A. Pokhrel S. Honermeier B. Schnell S. (2020). The response of the soil microbiota to long-term mineral and organic nitrogen fertilization is stronger in the bulk soil than in the rhizosphere. Genes (Basel) 11:456. doi: 10.3390/genes11040456, PMID: 32331348 PMC7230438

[ref2] Chen M. Q. Xu J. S. Li Z. Q. Li D. D. Wang Q. X. Zhou Y. P.. (2023). Long-term nitrogen fertilization-induced enhancements of acid hydrolyzable nitrogen are mainly regulated by the most vital microbial taxa of keystone species and enzyme activities. Sci. Total Environ. 874:162463. doi: 10.1016/j.scitotenv.2023.162463, PMID: 36842593

[ref3] Chen Q. Yuan Y. Hu Y. Wang J. Si G. Xu R.. (2021). Excessive nitrogen addition accelerates N assimilation and P utilization by enhancing organic carbon decomposition in a Tibetan alpine steppe. Sci. Total Environ. 764:142848. doi: 10.1016/j.scitotenv.2020.142848, PMID: 33268263

[ref4] Cheng H. Yuan M. Tang L. Shen Y. Yu Q. Li S. (2022). Integrated microbiology and metabolomics analysis reveal responses of soil microorganisms and metabolic functions to phosphorus fertilizer on semiarid farm. Sci. Total Environ. 817:152878. doi: 10.1016/j.scitotenv.2021.152878, PMID: 34998744

[ref5] Cui J. Yuan X. Zhang Q. Zhou J. Lin K. Xu J.. (2021). Nutrient availability is a dominant predictor of soil bacterial and fungal community composition after nitrogen addition in subtropical acidic forests. PLoS One 16:e0246263. doi: 10.1371/journal.pone.0246263, PMID: 33621258 PMC7901772

[ref6] Dai Z. Su W. Chen H. Barberán A. Zhao H. Yu M.. (2018). Long-term nitrogen fertilization decreases bacterial diversity and favors the growth of actinobacteria and proteobacteria in agro-ecosystems across the globe. Glob. Chang. Biol. 24, 3452–3461. doi: 10.1111/gcb.14163, PMID: 29645398

[ref7] Digby A. L. Gleason F. H. McGee P. A. (2010). Some fungi in the Chytridiomycota can assimilate both inorganic and organic sources of nitrogen. Fungal Ecol. 3, 261–266. doi: 10.1016/j.funeco.2009.11.002

[ref8] Du Y. Wang T. Wang C. Anane P. S. Liu S. Paz-Ferreiro J. (2019). Nitrogen fertilizer is a key factor affecting the soil chemical and microbial communities in a Mollisol. Can. J. Microbiol. 65, 510–521. doi: 10.1139/cjm-2018-0683, PMID: 30901528

[ref9] Fu Y. Liu T. Wang X. Wang Y. Gong Q. Li G.. (2023). Untargeted metabolomics reveal rhizosphere metabolites mechanisms on continuous ramie cropping. Front. Plant Sci. 14:1217956. doi: 10.3389/fpls.2023.1217956, PMID: 37674737 PMC10477603

[ref10] Hao T. Zhu Q. Zeng M. Shen J. Shi X. Liu X.. (2020). Impacts of nitrogen fertilizer type and application rate on soil acidification rate under a wheat-maize double cropping system. J. Environ. Manag. 270:110888. doi: 10.1016/j.jenvman.2020.110888, PMID: 32721326

[ref11] He D. Guo Z. M. Shen W. J. Ren L. J. Sun D. Yao Q.. (2022). Fungal communities are more sensitive to the simulated environmental changes than bacterial communities in a subtropical forest: the single and interactive effects of nitrogen addition and precipitation seasonality change. Microb. Ecol. 86, 521–535. doi: 10.1007/s00248-022-02092-8, PMID: 35927588

[ref12] He J. Jiao S. Tan X. Wei H. Ma X. Nie Y.. (2021). Adaptation of soil fungal community structure and assembly to long- versus short-term nitrogen addition in a tropical forest. Front. Microbiol. 12:689674. doi: 10.3389/fmicb.2021.689674, PMID: 34512567 PMC8424203

[ref13] He H. Qin J. Ma Z. Sun W. Yan W. He G.. (2020). Highly efficient regeneration and medicinal component determination of *Phellodendron chinense* Schneid. In Vitro Cell. Dev. Biol. Plant 56, 775–783. doi: 10.1007/s11627-020-10080-1

[ref14] He J. Tan X. Nie Y. Ma L. Liu J. Lu X.. (2023). Distinct responses of abundant and rare soil bacteria to nitrogen addition in tropical forest soils. Microbiol. Spectr. 11:e0300322. doi: 10.1128/spectrum.03003-22, PMID: 36622236 PMC9927163

[ref15] Huang C. Fu S. Ma X. Ma X. Ren X. Tian X.. (2023). Long-term intensive management reduced the soil quality of a Carya dabieshanensis forest. Sci. Rep. 13:5058. doi: 10.1038/s41598-023-32237-9, PMID: 36977743 PMC10050458

[ref16] Kim N. Riggins C. W. Zabaloy M. C. Rodriguez-Zas S. L. Villamil M. B. (2022). Limited impacts of cover cropping on soil N-cycling microbial communities of long-term corn monocultures. Front. Microbiol. 13:926592. doi: 10.3389/fmicb.2022.926592, PMID: 35755999 PMC9226624

[ref17] Lang M. Zou W. Chen X. Zou C. Zhang W. Deng Y.. (2020). Soil microbial composition and phoD gene abundance are sensitive to phosphorus level in a long-term wheat-maize crop system. Front. Microbiol. 11:605955. doi: 10.3389/fmicb.2020.605955, PMID: 33584568 PMC7873961

[ref18] Li C. Jia Z. Zhang S. Li T. Ma S. Cheng X.. (2023). The positive effects of mineral-solubilizing microbial inoculants on asymbiotic nitrogen fixation of abandoned mine soils are driven by keystone phylotype. Sci. Total Environ. 882:163663. doi: 10.1016/j.scitotenv.2023.163663, PMID: 37094687

[ref19] Li J. Li Y. Tian Y. Qu M. Zhang W. Gao L. (2017). Melatonin has the potential to alleviate cinnamic acid stress in cucumber seedlings. Front. Plant Sci. 8:1193. doi: 10.3389/fpls.2017.01193, PMID: 28751899 PMC5508022

[ref20] Li Y. Nie C. Liu Y. Du W. He P. (2019). Soil microbial community composition closely associates with specific enzyme activities and soil carbon chemistry in a long-term nitrogen fertilized grassland. Sci. Total Environ. 654, 264–274. doi: 10.1016/j.scitotenv.2018.11.031, PMID: 30445326

[ref21] Li Q. Yang J. He G. Liu X. Zhang D. (2022). Characteristics of soil C:N:P stoichiometry and enzyme activities in different grassland types in Qilian Mountain nature reserve-Tibetan Plateau. PLoS One 17:e0271399. doi: 10.1371/journal.pone.0271399, PMID: 35834549 PMC9282613

[ref22] Lian L. Lin Y. Wei Y. He W. Cai Q. Huang W.. (2021). PEPC of sugarcane regulated glutathione S-transferase and altered carbon-nitrogen metabolism under different N source concentrations in *Oryza sativa*. BMC Plant Biol. 21:287. doi: 10.1186/s12870-021-03071-w, PMID: 34167489 PMC8223297

[ref23] Liu M. Gan B. Li Q. Xiao W. Song X. (2022). Effects of nitrogen and phosphorus addition on soil extracellular enzyme activity and stoichiometry in chinese fir (*Cunninghamia lanceolata*) forests. Front. Plant Sci. 13:834184. doi: 10.3389/fpls.2022.834184, PMID: 35356128 PMC8959824

[ref24] Liu W. Jiang L. Yang S. Wang Z. Tian R. Peng Z.. (2020). Critical transition of soil bacterial diversity and composition triggered by nitrogen enrichment. Ecology 101:e03053. doi: 10.1002/ecy.3053, PMID: 32242918

[ref25] Liu H. Wang R. Lü X. T. Cai J. Feng X. Yang G.. (2021). Effects of nitrogen addition on plant-soil micronutrients vary with nitrogen form and mowing management in a meadow steppe. Environ. Pollut. 289:117969. doi: 10.1016/j.envpol.2021.117969, PMID: 34426201

[ref26] Liu M. Wei Y. Lian L. Wei B. Bi Y. Liu N.. (2023). Macrofungi promote SOC decomposition and weaken sequestration by modulating soil microbial function in temperate steppe. Sci. Total Environ. 899:165556. doi: 10.1016/j.scitotenv.2023.165556, PMID: 37459997

[ref27] Ma X. Song Y. Song C. Wang X. Wang N. Gao S.. (2021). Effect of nitrogen addition on soil microbial functional gene abundance and community diversity in permafrost peatland. Microorganisms 9:2498. doi: 10.3390/microorganisms9122498, PMID: 34946100 PMC8707234

[ref28] Navarro-León E. López-Moreno F. J. Borda E. Marín C. Sierras N. Blasco B.. (2022). Effect of l-amino acid-based biostimulants on nitrogen use efficiency (NUE) in lettuce plants. J. Sci. Food Agric. 102, 7098–7106. doi: 10.1002/jsfa.12071, PMID: 35778944 PMC9796150

[ref29] Nematollahi S. Pishdad G. R. Zakerkish M. Namjoyan F. Ahmadi Angali K. Borazjani F. (2022). The effect of berberine and fenugreek seed co-supplementation on inflammatory factor, lipid and glycemic profile in patients with type 2 diabetes mellitus: a double-blind controlled randomized clinical trial. Diabetol. Metab. Syndr. 14:120. doi: 10.1186/s13098-022-00888-9, PMID: 35999562 PMC9395822

[ref30] Peng Y. Chen G. Chen G. Li S. Peng T. Qiu X.. (2017). Soil biochemical responses to nitrogen addition in a secondary evergreen broad-leaved forest ecosystem. Sci. Rep. 7:2783. doi: 10.1038/s41598-017-03044-w, PMID: 28584271 PMC5459847

[ref31] Rauf A. Abu-Izneid T. Khalil A. A. Imran M. Shah Z. A. Emran T. B.. (2021). Berberine as a potential anticancer agent: a comprehensive review. Molecules 26:7368. doi: 10.3390/molecules26237368, PMID: 34885950 PMC8658774

[ref32] Si P. Shao W. Yu H. Xu G. Du G. (2022). Differences in microbial communities stimulated by malic acid have the potential to improve nutrient absorption and fruit quality of grapes. Front. Microbiol. 13:850807. doi: 10.3389/fmicb.2022.850807, PMID: 35663858 PMC9159917

[ref33] Song B. Li Y. Yang L. Shi H. Li L. Bai W.. (2023). Soil acidification under long-term N addition decreases the diversity of soil bacteria and fungi and changes their community composition in a semiarid grassland. Microb. Ecol. 85, 221–231. doi: 10.1007/s00248-021-01954-x, PMID: 35043220

[ref34] Sun Y. Lenon G. B. Yang A. W. H. (2019). Phellodendri cortex: a phytochemical, pharmacological, and pharmacokinetic review. Evid. Based Complement. Alternat. Med. 2019:7621929. doi: 10.1155/2019/7621929, PMID: 31057654 PMC6463642

[ref35] Sun J. Yang L. Wei J. Quan J. Yang X. (2020). The responses of soil bacterial communities and enzyme activities to the edaphic properties of coal mining areas in Central China. PLoS One 15:e0231198. doi: 10.1371/journal.pone.0231198, PMID: 32343698 PMC7188301

[ref36] Tang X. He Y. Zhang Z. Wu H. He L. Jiang J.. (2022). Beneficial shift of rhizosphere soil nutrients and metabolites under a sugarcane/peanut intercropping system. Front. Plant Sci. 13:1018727. doi: 10.3389/fpls.2022.1018727, PMID: 36531399 PMC9757493

[ref37] Tu L. H. Chen G. Peng Y. Hu H. L. Hu T. X. Zhang J.. (2014). Soil biochemical responses to nitrogen addition in a bamboo forest. PLoS One 9:e102315. doi: 10.1371/journal.pone.0102315, PMID: 25029346 PMC4100878

[ref38] Wang X. Fang L. Beiyuan J. Cui Y. Peng Q. Zhu S.. (2021). Improvement of alfalfa resistance against cd stress through rhizobia and arbuscular mycorrhiza fungi co-inoculation in cd-contaminated soil. Environ. Pollut. 277:116758. doi: 10.1016/j.envpol.2021.116758, PMID: 33652182

[ref39] Wang J. Shi X. Zheng C. Suter H. Huang Z. (2021). Different responses of soil bacterial and fungal communities to nitrogen deposition in a subtropical forest. Sci. Total Environ. 755:142449. doi: 10.1016/j.scitotenv.2020.142449, PMID: 33045514

[ref40] Wang Y.-F. Wang J.-F. Xu Z.-M. She S.-H. Yang J.-Q. Li Q.-S. (2020). L-glutamic acid induced the colonization of high-efficiency nitrogen-fixing strain Ac63 (*Azotobacter chroococcum*) in roots of *Amaranthus tricolor*. Plant Soil 451, 357–370. doi: 10.1007/s11104-020-04531-2

[ref41] Wang G. Wang J. Yao L. Li B. Ma X. Si E.. (2023). Transcriptome and metabolome reveal the molecular mechanism of barley genotypes underlying the response to low nitrogen and resupply. Int. J. Mol. Sci. 24:4706. doi: 10.3390/ijms24054706, PMID: 36902137 PMC10003240

[ref42] Wang L. Wen Y. Tong R. Zhang H. Chen H. Hu T.. (2022). Understanding responses of soil microbiome to the nitrogen and phosphorus addition in metasequoia glyptostroboides plantations of different ages. Microb. Ecol. 84, 565–579. doi: 10.1007/s00248-021-01863-z 34545413

[ref43] Want E. J. Masson P. Michopoulos F. Wilson I. D. Theodoridis G. Plumb R. S.. (2013). Global metabolic profiling of animal and human tissues via UPLC-MS. Nat. Protoc. 8, 17–32. doi: 10.1038/nprot.2012.135, PMID: 23222455

[ref44] Xiao J. Dong S. Shen H. Li S. Wessell K. Liu S.. (2022). N addition overwhelmed the effects of P addition on the soil C, N, and P cycling genes in Alpine Meadow of the Qinghai-Tibetan Plateau. Front. Plant Sci. 13:860590. doi: 10.3389/fpls.2022.860590, PMID: 35557731 PMC9087854

[ref45] Xu A. Li L. Xie J. Gopalakrishnan S. Zhang R. Luo Z.. (2022). Changes in ammonia-oxidizing archaea and bacterial communities and soil nitrogen dynamics in response to long-term nitrogen fertilization. Int. J. Environ. Res. Public Health 19:2732. doi: 10.3390/ijerph19052732, PMID: 35270425 PMC8910298

[ref46] Zhang H. Phillip F. O. Wu L. Zhao F. Yu S. Yu K. (2022). Effects of temperature and nitrogen application on carbon and nitrogen accumulation and bacterial community composition in apple rhizosphere soil. Front. Plant Sci. 13:859395. doi: 10.3389/fpls.2022.859395, PMID: 35444679 PMC9014127

[ref47] Zhang X. Song X. Wang T. Huang L. Ma H. Wang M.. (2022). The responses to long-term nitrogen addition of soil bacterial, fungal, and archaeal communities in a desert ecosystem. Front. Microbiol. 13:1015588. doi: 10.3389/fmicb.2022.1015588, PMID: 36312972 PMC9606763

[ref48] Zhang X. Sun W. Chen X. Chen L. Lv Z. He H.. (2023). Integrated physiological and transcriptomic analysis reveals mechanism of leaf in *Phellodendron Chinense* Schneid seedlings response to drought stress. Ind. Crop. Prod. 198:116679. doi: 10.1016/j.indcrop.2023.116679

[ref49] Zhang Z. Yu Z. Zhang Y. Shi Y. (2022). Impacts of fertilization optimization on soil nitrogen cycling and wheat nitrogen utilization under water-saving irrigation. Front. Plant Sci. 13:878424. doi: 10.3389/fpls.2022.878424, PMID: 35665172 PMC9161168

[ref50] Zhao Y. Yan C. Hu F. Luo Z. Zhang S. Xiao M.. (2022). Intercropping pinto peanut in litchi orchard effectively improved soil available potassium content, optimized soil bacterial community structure, and advanced bacterial community diversity. Front. Microbiol. 13:868312. doi: 10.3389/fmicb.2022.868312, PMID: 35633708 PMC9134032

