# Supplementary material for: The response of nutrient cycle, microbial community abundance and metabolic function to nitrogen fertilizer in rhizosphere soil of Phellodendron chinense Schneid seedlings
Source: Front Microbiol. 2023 Dec 15;14:1302775. doi: 10.3389/fmicb.2023.1302775 (PMC10762311; doi:10.3389/fmicb.2023.1302775)
Supplement: Supplementary file 1 [file Data_Sheet_1.pdf]

Supplementary Material

1 Supplementary Figures and Tables

1.1 Supplementary Tables

Supplementary Table 1 Primer sequences for PCR amplification

**Genes**		**Sequences** （5’→3’）
nifH	F	AAAGGYGGWATCGGYAARTCCACCAC
	R	TGSGCYTTGTCYTCRCGGATBGGCAT
phoD	F	CAGTGGGACGACCACGAGGT
	R	GAGGCCGATCGGCATGTCG
amoA-AOB	F	GGGGTTTCTACTGGTGGT
	R	CCCCTCKGSAAAGCCTTCTTC
nirk	F	ATYGGCGGVCAYGGCGA[a]
	R	GCCTCGATCAGRTTRTGG

Supplementary Table 2 Effects of N addition on relative abundance (%) of dominant bacterial phyla (Top 5) in rhizosphere soil of *P. chinense* Schneid seedlings

Treatments	*Proteobacteria*	*Acidobacteria*	*Bacteroidetes*	*Actinobacteria*	*Chloroflexi*
CK	40.73±2.62 b	26.39±1.84 a	5.22±0.32 b	3.26±0.36 b	5.17±0.21 a
N5	43.14±1.08 ab	23.92±0.89 a	6.12±0.40 b	3.96±0.24 b	4.26±0.26 b
N10	42.98±0.51 ab	23.51±0.80 a	7.09±0.54 ab	5.32±0.29 a	4.58±0.12 ab
N15	47.17±0.46 a	18.28±0.86 b	8.86±1.18 a	5.48±0.22 a	3.41±0.22 c

Data represent means±SD, n=3. The different letters following the numbers of the same parameter indicate significant difference (P<0.05). CK, control; N5, 5 g m^{-2}; N10, 10 g m^{-2}; N15, 15 g m^{-2}.

Supplementary Table 3 Effects of N addition on relative abundance (%) of dominant fungal phyla (Top 5) in rhizosphere soil of *P. chinense* Schneid seedlings

Treatments	*Basidiomycota*	*Ascomycota*	*Chytridiomycota*	*Mortierellomycota*	*Rozellomycota*
CK	60.12±3.18 a	24.87±1.48 b	0.26±0.05 b	0.94±0.37 a	0.23±0.14 b
N5	76.3±1.45 a	16.24±1.50 b	0.76±0.17 b	0.37±0.06 a	0.38±0.18 b
N10	66.53±4.98 a	22.58±4.12 b	2.44±0.53 b	0.76±0.15 a	0.12±0.01 b
N15	23.13±11.18 b	43.12±3.36 a	12.88±3.41 a	0.66±0.21 a	1.44±0.44 a

Data represent means±SD, n=3. The different letters following the numbers of the same parameter indicate significant difference ($P<0.05$). CK, control; N5, 5 g m^{-2}; N10, 10 g m^{-2}; N15, 15 g m^{-2}.

1.2 Supplementary Figures

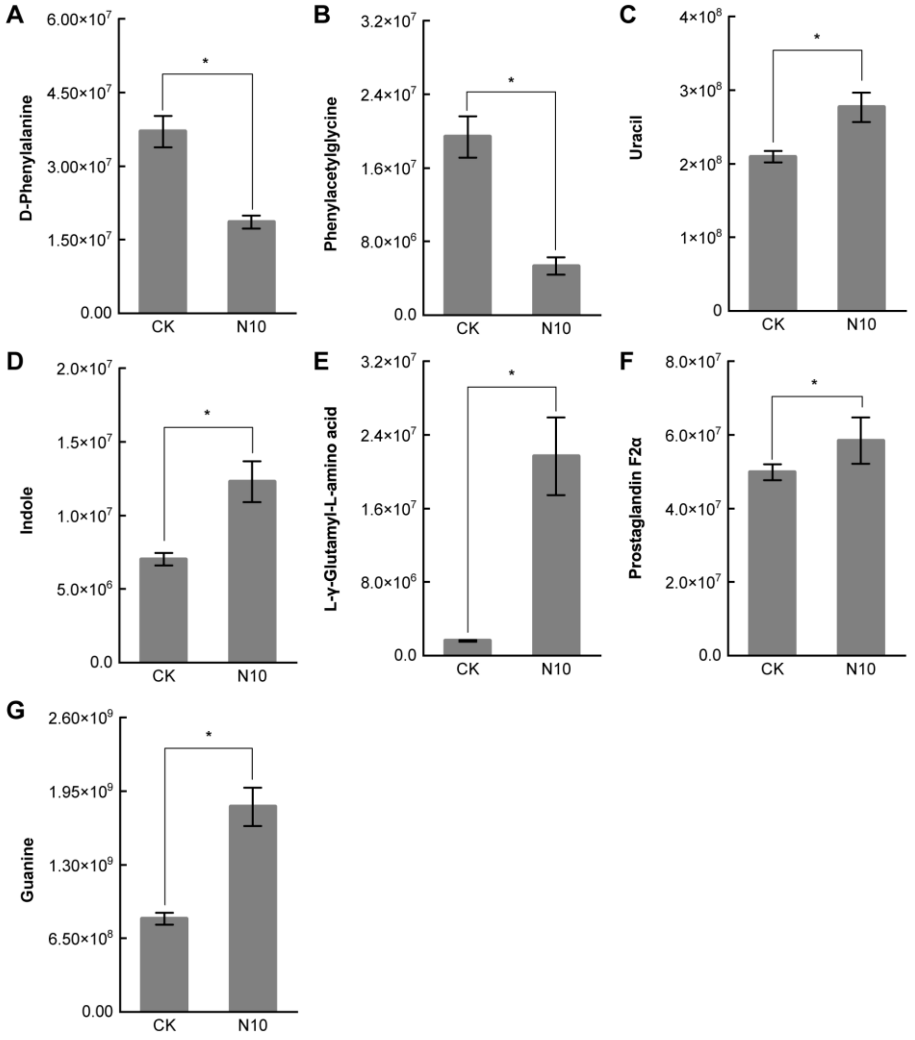

Supplementary Figure 1. Effects of N10 on the absolute abundances of DAMs in rhizosphere soil of *P. chinense* Schneid seedlings. The single asterisks on the bar indicate significant difference ($P<0.05$). CK, control; N10, 10 g m^{-2}.

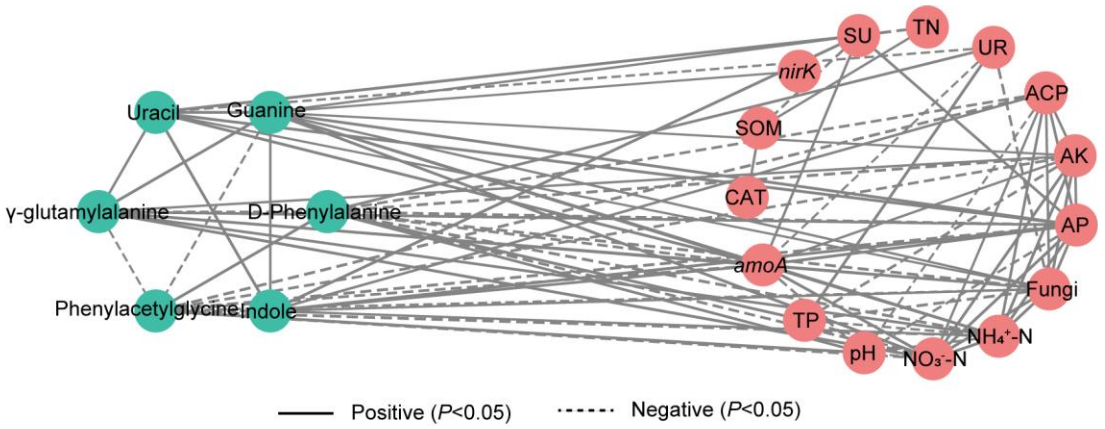

Supplementary Figure 2. Correlation analysis of soil physicochemical properties, relative expression abundance of functional genes, microbial diversity and DAMs under N10 treatment. Only the indicators that have a significant correlation are shown in the figure. SOM, soil organic matter; TN, total nitrogen; TP, total phosphorus; AP, available phosphorus; AK, available potassium; CAT, catalase; SU, sucrase; ACP, acid phosphatase; UR, urease.
